# Cerevicalgia and Depression: Comorbidity and Pathopysiology

**DOI:** 10.1192/j.eurpsy.2025.1206

**Published:** 2025-08-26

**Authors:** D. Labunskiy, N. Kurgaev, D. Baranov, S. Kiryukhina, D. Kuzmin

**Affiliations:** 1 Ogarev Mordovia State University, Saransk; 2 Vidnoe Hospital, Vidnoe, Russian Federation

## Abstract

**Introduction:**

The widespread prevalence of neck pain does not mean that one should stoically endure suffering. There is some truth in the fact that many people have such problems, but it is not true that one should not go to the doctor for such trivial reasons. Therefore, if pain in neck occurs, you should go to a neurologist immediately. This is especially true in cases where episodes of pain are repeated, they are persistent or intensify.

**Objectives:**

There are many causes of neck pain:

Fractures, dislocations, injuries to the vertebrae, joints, muscles and ligaments in the neck.

Infections that cause inflammation of bone and cartilage tissue, muscles, cervical lymph nodes and nerves (osteomyelitis, abscess, shingles).

Radiating pain in diseases of the heart, lungs, stomach, esophagus, and other organs. Rheumatological diseases (arthritis, myalgia, fibromyalgia).

**Methods:**

Statistical analysis was performed.

**Results:**

Neuroendocrine pathologies, non-specific factors (herniated and displaced intervertebral discs, problems with intervertebral joints, narrowing of the spinal canal, arthrosis, muscle spasms, osteochondrosis, problems caused by insufficient muscle activity), Intracranial diseases (benign or malignant neoplasms of the brain, stroke, abscess), and also psychosomatics.

**Conclusions:**

There are several reasons that cause depression due to depletion of cartilage tissue. First of all, we are talking about the disruption of cerebral circulation associated with disorders in the cervical spine. Lack of blood flow, and therefore oxygen, leads to starvation of various parts of the brain responsible for certain important functions of the body. This is how nervous system disorders begin to develop: depression, aggression, panic attacks and insomnia.

**Disclosure of Interest:**

None Declared

